# MOF-Derived In_2_O_3_-CeO_2_ Composite Catalyst with Abundant Oxygen Vacancies for Photothermal CO_2_ Reduction

**DOI:** 10.3390/nano16140872

**Published:** 2026-07-15

**Authors:** Huiqing Dong, Siyu Huang, Haopeng Cui, Ziyi Zhang, Dongxu Zhou, Weikai Huang, Xiaodong Zhang, Zhongxiao Zhang, Jianqiu Lei, Ning Liu

**Affiliations:** 1 School of Environment and Architecture, University of Shanghai for Science and Technology, Shanghai 200093, China; dhq200223@163.com (H.D.); 13958318481@163.com (S.H.); m15930993445@163.com (H.C.); 18067069811@163.com (Z.Z.); zdx17730040824@163.com (D.Z.); 15021163085@163.com (W.H.); fatzhxd@126.com (X.Z.); 2 Beijing National Laboratory for Molecular Sciences, CAS Key Laboratory of Colloid, Interface and Chemical Thermodynamics, Institute of Chemistry, Chinese Academy of Sciences, Beijing 100190, China; 3 Shanghai Noncarbon Energy Conversion and Utilization Institute, Shanghai 200240, China; zhzhx222@163.com; 4 College of Smart Energy, Shanghai Jiao Tong University, Shanghai 200240, China; 5 Shanghai Institute of Optics and Fine Mechanics, Chinese Academy of Sciences, Shanghai 201800, China; ljqlei@163.com

**Keywords:** photothermal catalysis, CO_2_ hydrogenation, In_2_O_3_-CeO_2_, oxygen vacancies

## Abstract

The conversion of CO_2_ into value-added chemicals using photothermal catalysis is an attractive approach for achieving carbon neutrality. However, the limited visible-light absorption and rapid charge carrier recombination of CeO_2_ hinder its photothermal catalytic performance. Herein, a series of In_2_O_3_-modified CeO_2_ (In_2_O_3_-CeO_2_) are fabricated from a Ce-BTC metal–organic framework (MOF) precursor and applied to photothermal CO_2_ hydrogenation. This approach not only effectively modulates the pore structure but also generates abundant oxygen vacancies (O_v_). As a result, CO_2_ adsorption and activation, charge separation and photothermal catalytic performance over In_2_O_3_-CeO_2_ are significantly enhanced. The 3% In_2_O_3_-CeO_2_ (3% denotes amounts of In) composite catalyst exhibits the optimal catalytic performance with a CO yield of 92.35 mmol·g^−1^·h^−1^ and a remarkable 100% selectivity. A mechanistic study reveals that the Ov-rich interface facilitates electron transfer and promotes CO_2_ conversion through a CO_2_ → *COOH → *CO reaction pathway. This work provides an effective strategy for designing high-efficiency MOF-derived composites to achieve carbon utilization.

## 1. Introduction

To realize global carbon neutrality, the efficient capture and removal of CO_2_ into value-added chemicals or fuels is essential for sustainable development [1,2]. CO_2_ hydrogenation has attracted significant attention due to its capacity to produce industrial feedstocks such as CO, methane, and methanol [3,4]. In particular, the reverse water–gas shift (RWGS) reaction stands out as a highly promising route. However, the strong C=O bond in CO_2_ (≈799 kJ·mol^−1^) provides it with high thermodynamic stability, and photocatalysis is often limited by low quantum efficiency [5]. Therefore, photothermal catalysis has garnered attention as a highly potential strategy that integrates the advantages of thermal catalysis and photocatalysis, providing an effective pathway to overcome the activation barrier of CO_2_ [6,7].

CeO_2_ is widely recognized as an ideal material for photothermal CO_2_ reduction due to its outstanding redox properties and exceptional oxygen storage capacity (OSC) [8,9]. To further optimize its performance, a metal–organic framework (MOF) derivation strategy using Ce-BTC as a precursor can be employed [10]. This approach fully utilizes the resulting tunable pore structures and spatial confinement effects, making the MOF-derived CeO_2_ an excellent support for stabilizing various metal-based catalysts [11,12]. Upon high-temperature calcination, the resulting CeO_2_ develops highly dispersed active sites and abundant oxygen vacancies (O_v_) at the interface, thereby significantly enhancing the adsorption and activation of CO_2_ [13]. As an excellent catalyst for CO_2_ conversion, In_2_O_3_ can leverage its n-type semiconducting characteristics to expand visible-light harvesting and suppress charge recombination [14]. Paramita Koley et al. designed In_2_O_3_/ZrO_2_ heterojunction exhibiting abundant O_v_ at the interface and enhancing the catalytic performance [15]. Yang and co-workers fabricated a Z-scheme heterojunction by anchoring In_2_O_3_ onto CuGa_0.5_S via a facile ball-milling approach, enabling the highly selective and sacrificial-free conversion of CO_2_ to CH_4_ [16]. Moreover, in the study of Oliver Martin et al., In_2_O_3_/ZrO_2_ has demonstrated with high activity and selectivity for methanol synthesis by CO_2_ hydrogenation [17]. Furthermore, it was demonstrated by Wang et al. that the RWGS catalytic performance of In_2_O_3_-CeO_2_ was enhanced while maintaining 100% CO selectivity [18]. This synergy is likely rooted in the strong interaction at the In_2_O_3_-CeO_2_ interface, which promotes O_v_ formation and stabilizes key reaction intermediates. However, a comprehensive understanding from Ce-MOF to the active In_2_O_3_-CeO_2_ interface remains to be explored.

In this study, we developed a composite catalyst In_2_O_3_-CeO_2_ providing abundant O_v_ for CO_2_ activation, and In_2_O_3_ enhanced H_2_ dissociation. Furthermore, the synergistic effect between In_2_O_3_ and CeO_2_ significantly promotes the formation and stabilization of *COOH. Our results demonstrate that the composite catalyst 3%In_2_O_3_-CeO_2_ exhibits superior CO production and selectivity compared to CeO_2_, offering a promising strategy for efficient CO_2_-to-CO conversion.

## 2. Experimental Section

### 2.1. Chemicals

All chemical reagents were acquired from commercial sources and utilized as received without further purification. Specifically, cerium (III) nitrate hexahydrate (Ce(NO_3_)_3_·6H_2_O, 99.99%) and 1,3,5-benzenetricarboxylic acid (BTC, 99%+) were purchased from Aladdin Bio-Chem Technology Co., Ltd. (Shanghai, China). Indium (III) nitrate pentahydrate (In(NO_3_)_3_·5H_2_O, 99.9%) was supplied by Titan Scientific Co., Ltd. (Shanghai, China). Methanol (≥99.7%) and ethanol (≥99.7%) were provided by Sinopharm Chemical Reagent Co., Ltd. (Shanghai, China).

### 2.2. Preparation of Catalysts

#### 2.2.1. Synthesis of Ce-BTC

The Ce-BTC precursor was synthesized by dissolving both of 1.5 mmol Ce (NO_3_)_3_·6H_2_O and BTC in a 30 mL methanol and 30 mL ethanol. Following 5 min of rapid stirring, 30 mL of deionized (DI) water was added to the mixture. The mixture was maintained under stirring for 20 min. Ce-BTC was then collected via centrifugation at 5500 rpm for 5 min, washed thoroughly with ethanol, and vacuum-dried at 70 °C for 12 h.

#### 2.2.2. Synthesis of xIn_2_O_3_-CeO_2_ Catalyst

**Figure 1:**
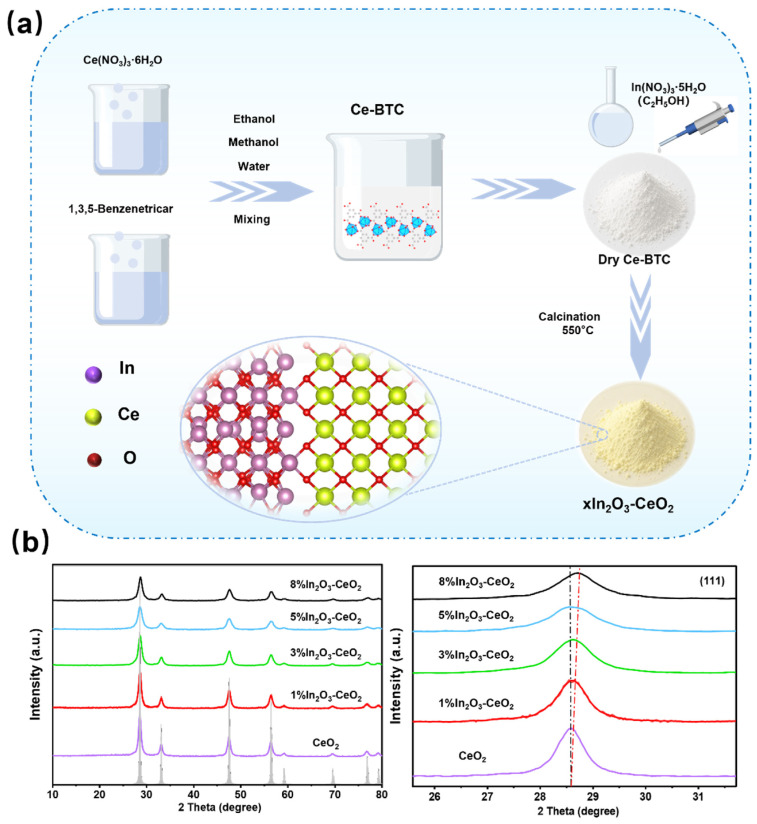
(**a**) Synthesis scheme of xIn_2_O_3_-CeO_2_. (**b**) XRD patterns and partially magnified profiles of the CeO_2_ and xIn_2_O_3_-CeO_2_.

In total, 1.0 g In(NO_3_)_3_·5H_2_O was dissolved in ethanol and diluted to 100 mL to prepare a 10 mg mL^−1^ precursor solution. Subsequently, a specific volume of the mother liquor was measured and added to the Ce-BTC by the wet impregnation method. Following a 12 h drying process at 70 °C, the dry powder was subjected to thermal calcination at 550 °C for 4 h, employing a 2 °C min^−1^ under an ambient air flow. The system was then allowed to cool to room temperature, and the samples were collected as xIn_2_O_3_-CeO_2_ (x = 1%, 3%, 5%, and 8%). The specific amounts of In (NO_3_)_3_·5H_2_O and precursor solution are summarized in Table S1. The detailed process is illustrated in Figure 1a.

### 2.3. Catalytic Activity Evaluation

High-purity gas reagents (>99.99 vol%) were used without further treatment. Photothermal catalytic tests were conducted in a Beijing Perfect-light PLR-RVTF-POB fixed-bed reactor under a PLS-SXE^+^(III) Xenon lamp. For each catalytic measurement, 100 mg of the active catalyst was dispersed within a 300 mg quartz sand. The reactant mixture (CO_2_:H_2_:Ar = 1:1:2) was introduced at 40–80 mL min^−1^. Following a 30 min at 0.1 MPa to remove impurities, the bed temperature was maintained between 200 and 500 °C. Product distribution (CO_2_, CO and CH_4_) was determined using an online GC-FID system.

The products selectivity (S, %), CO_2_ conversion (X_CO_2__, %), and production formation rate (r) are calculated according to the following equations:

(1) $$S_{CO}\;=\;\frac{n_{CO}^{out}}{n_{CO}^{out}+n_{{CH}_{4}}^{out}}\;\times \;100\%$$

(2) $$S_{{\mathrm{CH}}_{4}}=\frac{n_{{CH}_{4}}^{out}}{n_{CO}^{out}+n_{{CH}_{4}}^{out}}\times 100\%$$

(3) $$X_{{\mathrm{CO}}_{2}}=\frac{{CO}_{2}^{in}-{CO}_{2}^{out}}{{CO}_{2}^{in}}\times 100\%$$

(4) $$R=\frac{S_{co}\times X{co}_{2}\times F{co}_{2}^{in}}{m_{cat}\times V_{m}}\times 60$$

where *n*_CO_*^out^* and *n*_CH4_*^out^* represent the molar amounts of CO and CH_4_ in the outlet gas; CO_2_*^in^* and CO_2_*^out^* are the CO_2_ molar numbers before or after the reaction; *F*_CO2_*^in^* represents the gas flow rate (mL⋅min^−1^) of CO_2_; *m_cat_* represents the mass (mg) of catalyst; *V_m_* represents 22.4 L mol^−1^.

### 2.4. Catalyst Characterizations

The crystal structures of the prepared samples were characterized by powder X-ray diffraction (XRD). XRD patterns of the catalysts were recorded on a Bruker D8 ADVANCE diffractometer using Cu Kα radiation (λ = 0.15418 nm) as the X-ray source. Morphological features and elemental compositions were investigated using a Philips FEI XL-30 scanning electron microscope (SEM) coupled with energy-dispersive X-ray spectroscopy (EDS). An inductively coupled plasma optical emission spectroscope (ICP-OES, Agilent 5100, Santa Clara, CA, USA) was employed to determine the actual In content of the catalysts. Before measurement, the solid catalyst samples were pretreated by microwave-assisted acid digestion. TEM (Transmission Electron Microscope) and HRTEM (High-ResolutionTransmission Electron Microscope) images were obtained using an FEI Talos F200S transmission electron microscope operated at 200 kV. The textural properties of the catalysts, including the Brunauer–Emmett–Teller (BET) specific surface areas and Barrett–Joyner–Halenda (BJH) pore size distributions, were determined on a Quantachrome Autosorb-iQ-2MP instrument via N_2_ adsorption–desorption isotherms at 77 K following degassing at 200 °C for 4 h. Fourier-transform infrared (FT-IR) spectra were collected on a Thermo Fisher Scientific Nicolet iS50 spectrometer within 4000–400 cm^−1^ using the KBr pellet technique. Raman scattering spectra were acquired on a HORIBA Scientific LabRAM HR Evolution confocal Raman spectrometer using a 532 nm excitation laser. A UV-vis diffuse reflectance spectroscope (UV-vis DRS, UV-2600, Shimadzu Corporation, Kyoto, Japan) was employed to probe optical properties in the range of 200–800 nm, utilizing BaSO_4_ as the white standard. X-ray Photoelectron Spectroscopy (XPS) analysis was performed on an ESCALAB 250Xi instrument, with all binding energies referenced to the C 1s peak at 284.8 eV. Steady-state photoluminescence (PL) spectra and time-resolved photoluminescence (TRPL) decay curves were recorded using an Edinburgh Instruments FS5 spectrofluorometer. Finally, in situ DRIFTS was performed on a 300 W Xenon lamp and monitored the photothermal CO_2_ reduction (100–500 °C, 800–4000 cm^−1^ under a mixed flow (CO_2_/H_2_/Ar = 10/10/20 mL/min, 0.1 MPa) after Ar pretreatment at 300 °C.

## 3. Results and Discussion

### 3.1. Catalyst Characterization

The crystalline structures of Ce-BTC, CeO_2_, and xIn_2_O_3_-CeO_2_ were characterized by XRD. The diffraction pattern of Ce-BTC is consistent with previously reported results, confirming its successful synthesis (Figure S2a) [19]. As shown in Figure 1b, the Ce-BTC-derived CeO_2_ exhibits characteristic diffraction peaks at 28.5°, 33.0°, 47.5°, and 56.3°, which can be indexed to the (111), (200), and (311) crystal planes of cubic CeO_2_ (PDF#34-0394), indicating its high crystallinity and confirming the successful synthesis of CeO_2_ [20]. The main diffraction peaks of xIn_2_O_3_-CeO_2_ remain the main peaks of CeO_2_. Notably, the intensities of the diffraction peaks ascribed to CeO_2_ vary with increasing In_2_O_3_ content. As the In_2_O_3_ concentration increases, the CeO_2_ (111) peaks become smaller and broader, accompanied by a slight shift toward the higher diffraction angles, suggesting that the introduction of In influences the crystal structure of CeO_2_. This phenomenon is ascribed to the partial replacement of Ce^4+^ by In^3+^ within the CeO_2_ crystal lattice, where the difference in ionic size leads to a decrease in the lattice parameters of CeO_2_ [21].

**Figure 2:**
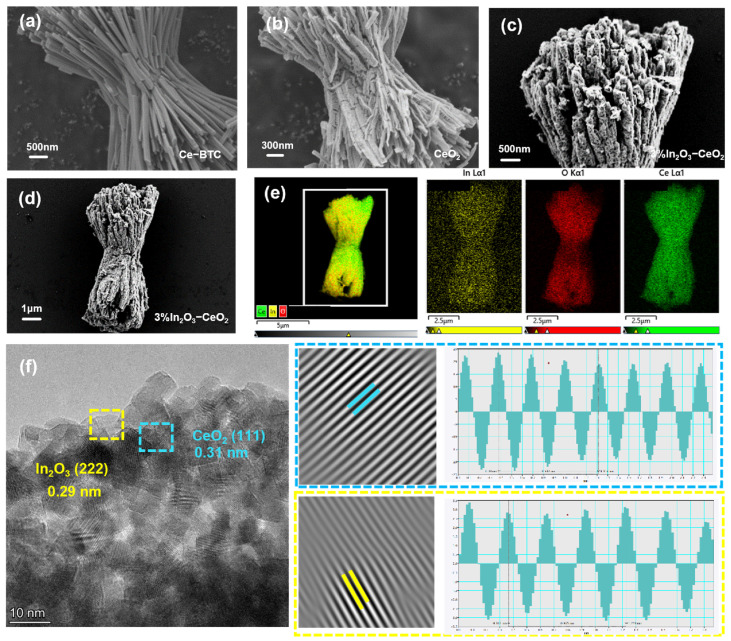
SEM images of (**a**) Ce-BTC, (**b**) CeO_2_, and (**c**,**d**) 3% In_2_O_3_-CeO_2_. (**e**) EDS mapping images of 3% In_2_O_3_-CeO_2_. (**f**) HRTEM images and IFFT crystalline lattice images with the line intensity profile of 3% In_2_O_3_-CeO_2_.

The morphological features of the prepared catalysts were scrutinized via SEM. As shown in Figure 2a, Ce-BTC exhibits a rod-like structure with a smooth surface. Ce-BTC derived CeO_2_ keeps the original morphology of Ce-BTC, and its surface becomes rough (Figure 2b). After the loading of In, 3%In_2_O_3_-CeO_2_ still maintains the rod-like structure (Figure 2c,d), but numerous voids appear on its surface, mainly stemming from the degradation of organic linkers during the high-temperature calcination process. The corresponding EDS elemental mapping results of 3%In_2_O_3_-CeO_2_ clearly demonstrate the successful introduction and uniform distribution of In species on CeO_2_ (Figure 2e and Table S2). The In content calculated from EDS mapping is 1.88 wt%, while ICP-OES analysis after microwave-assisted digestion gives 2.36 wt% for 3%In_2_O_3_-CeO_2_, as listed in Table S3. The corresponding calculation formula for ICP-OES can be found in the supporting information. TEM and HRTEM analyses further investigated the microstructure of 3%In_2_O_3_–CeO_2_. As observed from the TEM images in Figure S3a, 3%In_2_O_3_–CeO_2_ retains a distinct rod-like morphology. The HRTEM image in Figure 2f displays clear and continuous lattice fringes. The lattice spacing of 0.31 nm can be assigned to the (111) plane of CeO_2_, while the spacing of 0.29 nm is consistent with the (222) plane of In_2_O_3_ [22].

**Figure 3:**
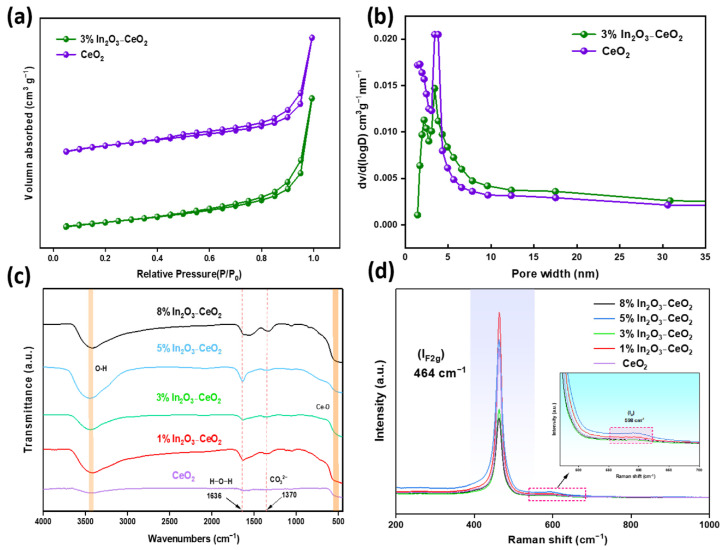
(**a**) N_2_ adsorption–desorption isotherms, (**b**) Pore size distribution of CeO_2_ and 3%In_2_O_3_-CeO_2_. (**c**) FT-IR and (**d**) Raman spectra of CeO_2_ and xIn_2_O_3_-CeO_2_.

The pore structure properties of the catalysts were evaluated using N_2_ adsorption–desorption isotherms. As shown in Figure S2b,c, the Ce-BTC displays a typical Type I isotherm, implying its characteristic microporous structure. In contrast, both CeO_2_ and 3%In_2_O_3_-CeO_2_ exhibit Type IV isotherms, indicating the formation of mesoporous structure after calcination (Figure 3a). The pore size distributions further confirm the distinct structural transformation from the microporous Ce-BTC to the mesoporous CeO_2_ and 3%In_2_O_3_-CeO_2_ (Figure 3b). The specific surface areas (S_BET_) and pore parameters of the catalysts are summarized in Table S3. Compared to CeO_2_, the S_BET_ of 3% In_2_O_3_-CeO_2_ slightly decreased from 111.429 m^2^/g to 83.794 m^2^/g, which is primarily attributed to the interfacial occupation of In_2_O_3_ on the CeO_2_ [23]. However, the pore volume increased from 0.262 to 0.285 cc/g, indicating the formation of a more open porous structure. The increased pore volume facilitates CO_2_ diffusion and mass transfer during the catalytic reaction, allowing CO_2_ to access the active sites more efficiently. This improved pore structure is therefore favorable for enhancing reactant transport and accelerating the catalytic process [24]. 

FT-IR spectra is an effective method for verifying the functional group structure of materials. The FT-IR spectra of xIn_2_O_3_-CeO_2_ and CeO_2_ are shown in Figure 3c. The absorption peaks of xIn_2_O_3_-CeO_2_ are similar with that of CeO_2_. However, compared with CeO_2_, xIn_2_O_3_-CeO_2_ exhibit slightly enhanced peak intensities, indicating the successful introduction of In species. The absorption band below approximately 650 cm^−1^ is attributed to the Ce–O lattice vibrations, while the broad band centered at around 3420 cm^−1^ originates from the O–H stretching vibrations of surface hydroxyl groups and adsorbed water [25]. The band at 1636 cm^−1^ is assigned to the H–O–H bending vibration of molecularly adsorbed water, whereas the band at 1370 cm^−1^ is associated with surface carbonate species formed by atmospheric CO_2_ adsorption on the basic sites of CeO_2_ and xIn_2_O_3_–CeO_2_ [26].

Raman spectroscopy was further employed to elucidate the structural characteristics of the catalyst. For Ce-BTC, the prominent spectral features at 1566 and 1451 cm^−1^ signify the asymmetric and symmetric vibrations of carboxylate linkers, respectively (Figure S2d) [27]. For CeO_2_ and xIn_2_O_3_-CeO_2_, the distinct peaks at 400 and 246 cm^−1^ are associated with the lattice vibrations of Ce-O and Ce-Ce. Furthermore, a distinct F_2g_ peak centered at 464 cm^−1^ is observed, which is characteristic of the fluorite structure of CeO_2_ (Figure 3d) [28]. In addition, a broad peak observed at 570–600 cm^−1^ signifies a defect-induced Raman mode, directly confirming the introduction of O_v_ upon In^3+^ doping [29]. The relative concentration of O_v_ is evaluated by the intensity ratio of the defect peak to the F_2g_ peak, and the 3% In_2_O_3_-CeO_2_ exhibits the highest O_v_ concentration among all the prepared catalysts.

**Figure 4:**
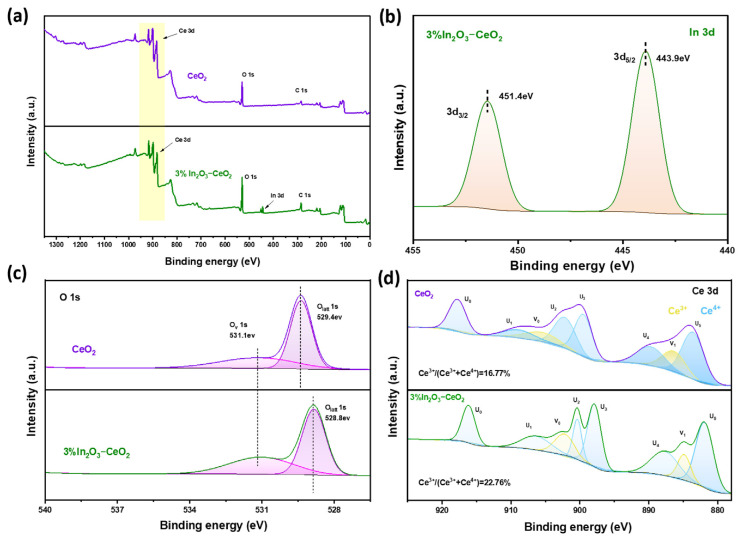
The XPS spectra of CeO_2_ and 3%In_2_O_3_-CeO_2_: (**a**) survey, (**b**) In 3d of 3%In_2_O_3_-CeO_2_, (**c**) O 1 s, and (**d**) Ce 3d.

The surface electronic and chemical states of each element were analyzed via XPS. As shown in Figure 4a, the survey spectrum clearly shows the coexistence of Ce, In, and O elements, further confirming the introduction of In species in 3% In_2_O_3_-CeO_2_. The surface elemental composition is further quantitatively analyzed in the supporting information. As summarized in Table S5, the In content was calculated to be 1.40 at.% after excluding carbon contamination. Combined with the EDS mapping and ICP-OES results, the XPS result verifies that In species were successfully introduced into CeO_2_. In Figure 4b, the In 3d spectrum of 3% In_2_O_3_-CeO_2_ exhibits two distinct peaks corresponding to In^3+^ 3d_3/2_ and In^3+^ 3d_5/2_, suggesting that In mainly exists in the form of In^3+^ [30], which is consistent with the XRD analysis. The O 1s XPS spectra in Figure 4c can be deconvoluted into two primary peaks, including lattice oxygen (O_latt_) located at 528.8–529.4 eV and O_v_ at 531.1 eV. Compared to CeO_2_, 3% In_2_O_3_-CeO_2_ exhibits a higher proportion of O_v_ species, indicating that the introduction of In promotes the generation of O_v_. Meanwhile, the O_latt_ peak shifted significantly toward a lower binding energy by 0.6 eV from 529.4 eV to 528.8 eV after In incorporation. This reflects a charge transfer from In species to the CeO_2_ lattice, which alters the electronic environment around the oxygen atoms [31]. In Figure 4d, the Ce 3d spectrum can be well resolved into eight distinct peaks, which are successfully assigned to the Ce 3d_5/2_ and Ce 3d_3/2_ spin–orbit components. The two peak “v-lines” belong to Ce^3+^ and the six “u-lines” are ascribed to Ce^4+^. More importantly, the Ce^3+^ content in 3% In_2_O_3_-CeO_2_ increased from 16.77% to 22.76% compared with CeO_2_, representing a higher concentration of O_v_ [32]. And in 3% In_2_O_3_-CeO_2_, the binding energy of Ce 3d shifts toward lower values, implying a strong interaction between In and Ce. This interaction alters the electron density of Ce and leads to the downward shift in its binding energy [33]. Overall, 3% In_2_O_3_-CeO_2_ possesses a higher concentration of O_v_ and Ce^3+^. These features are beneficial for CO_2_ adsorption and activation, thereby contributing to the enhanced photothermal RWGS performance of the 3% In_2_O_3_-CeO_2_ composite catalyst.

### 3.2. Catalytic Performance Evaluation

**Figure 5:**
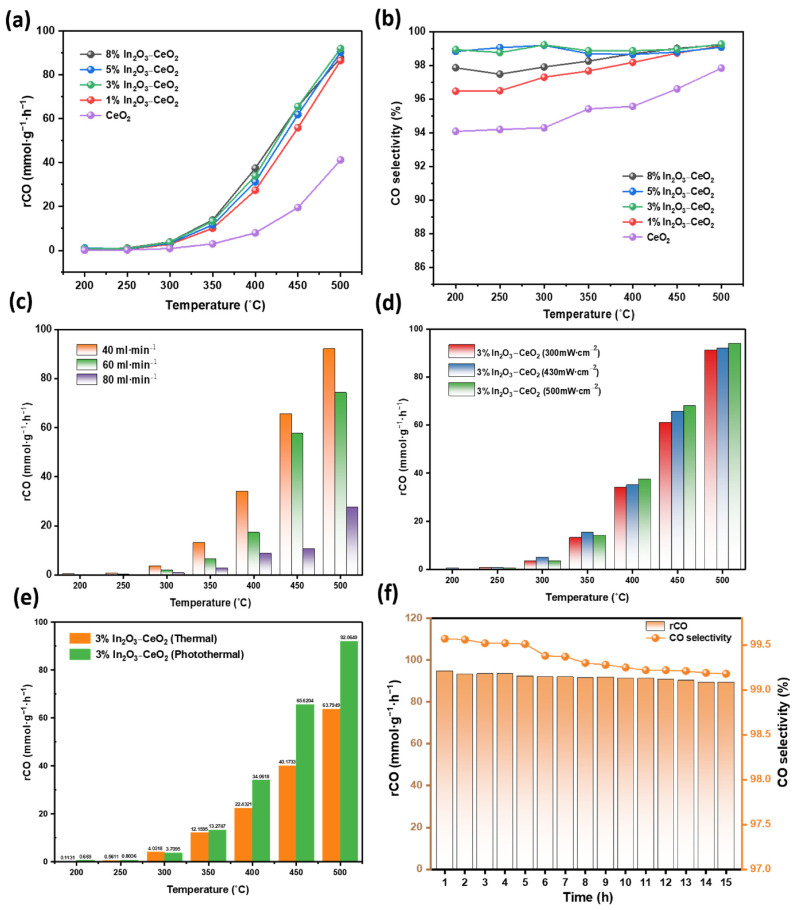
Catalytic performance of CeO_2_ and xIn_2_O_3_-CeO_2_ for CO_2_ photothermal reaction. (**a**) CO production rates, (**b**) CO selectivity over CeO_2_ and xIn_2_O_3_-CeO_2_. (**c**) CO production rates over xIn_2_O_3_-CeO_2_ at different gas flow. (**d**) CO production rates over xIn_2_O_3_-CeO_2_ under different light intensities. (**e**) CO production rates over xIn_2_O_3_-CeO_2_ under photothermal or thermal conditions. (**f**) Production rates and selectivity of CO over xIn_2_O_3_-CeO_2_ during 15 h reaction.

Figure 5 presents the photothermal catalytic CO_2_ reduction performance of CeO_2_ and xIn_2_O_3_-CeO_2_. As shown in Figure 5a, CO is the main product over CeO_2_ and xIn_2_O_3_-CeO_2_ during photothermal CO_2_ reduction. Among these catalysts, the 3% In_2_O_3_-CeO_2_ catalyst exhibits the highest CO production rate of 92.35 mmol·g^−1^·h^−1^. Figure 5b further illustrates that 3%In_2_O_3_-CeO_2_ has superior CO selectivity compared with the other catalysts, maintaining nearly 100% selectivity over the temperature range from 200 to 500 °C. As presented in Figure S4, the CO_2_ conversion on 3%In_2_O_3_–CeO_2_ reaches 30.13%. The catalytic performances under different flow rates (40, 60 and 80 mL·min^−1^) are compared in Figure 5c. The highest CO yield is achieved at a total flow rate of 40 mL·min^−1^, suggesting that an appropriate residential time of reaction gases is beneficial for the photothermal catalytic reaction. Figure 5d compares the photothermal performance of 3%In_2_O_3_-CeO_2_ under varying light intensities. The CO yield increases with increasing light intensity, demonstrating the positive contribution of light to the photothermal process. However, when the light intensity increases from 430 mW cm^−2^ to 500 mWcm^−2^, only a slight improvement in the CO yield is observed at 500 °C. Consequently, considering the energy efficiency ratio and economic feasibility, a light intensity of 430 mWcm^−2^ is selected as the standard condition for subsequent experiments. To quantify the synergy of light and heat, comparative experiments were conducted under both photothermal and thermal catalytic conditions (Figure 5e). It is clear that the CO_2_ reduction efficiency in photothermal catalysis is significantly higher than that in the thermal catalytic condition. The light contribution was estimated using $\mathrm{light}\;\mathrm{contribution}=(r_{\mathrm{photothermal}}-r_{\mathrm{thermal}})/r_{\mathrm{thermal}}\times 100\%$, giving values of 34.1%, 38.8%, and 30.7% at 400, 450, and 500 °C, respectively. These results demonstrate that light irradiation makes a considerable contribution beyond the pure thermal effect. To further evaluate the photothermal response of the catalyst, the catalyst-bed temperature evolution of 3%In_2_O_3_-CeO_2_ under light irradiation was recorded. As shown in Figure S5, the temperature of the catalyst bed increases rapidly after light illumination and gradually reaches a stable value. The initial temperature at 0 s represents the dark state before light irradiation. The obvious temperature increase under illumination confirms the effective photothermal conversion ability of 3%In_2_O_3_-CeO_2_. Finally, a 15 h continuous stability test was performed over 3%In_2_O_3_-CeO_2_ (Figure 5f). No obvious decrease in catalytic activity was determined during the photothermal process, demonstrating the excellent chemical stability and structural robustness of 3%In_2_O_3_-CeO_2_ under high-temperature photothermal conditions. In addition, we compared the CO production performance and selectivity of 3%In_2_O_3_-CeO_2_ with those of representative CeO_2_-based catalysts. As summarized in Table S6, 3%In_2_O_3_-CeO_2_ exhibits a superior CO production rate of 92.35 mmol g^−1^ h^−1^ with nearly 100% CO selectivity, suggesting its highly competitive photothermal CO_2_-to-CO conversion [34,35,36,37,38,39].

### 3.3. Mechanism of Photothermal Catalysis

**Figure 6:**
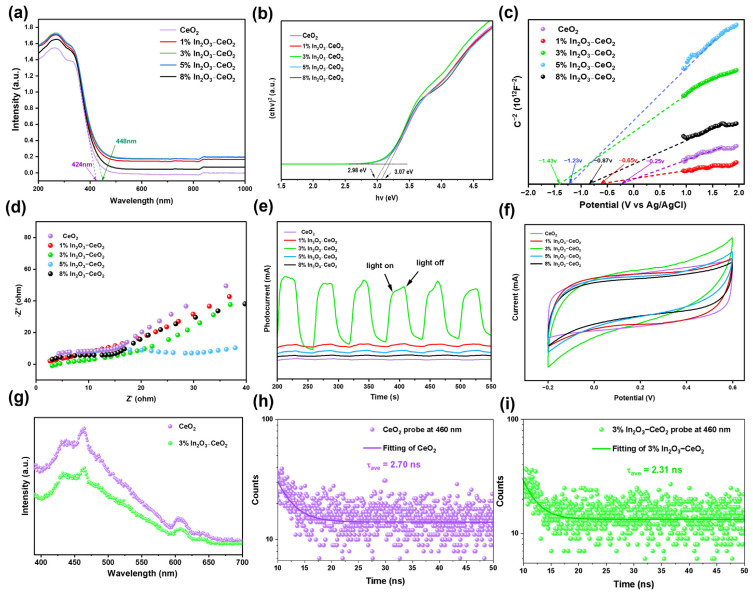
(**a**) UV-vis DRS spectra, (**b**) Band gap, (**c**) Mott–Schottky curve, (**d**) EIS Nyquist plots, (**e**) Photocurrent responses, (**f**) CV curve of CeO_2_ and xIn_2_O_3_-CeO_2_. (**g**) PL spectra of CeO_2_ and 3%In_2_O_3_-CeO_2_. (**h**) TRPL of CeO_2_. (**i**) TRPL of 3%In_2_O_3_-CeO_2_.

The optical absorption behaviors and electronic band configurations of CeO_2_ and xIn_2_O_3_-CeO_2_ were investigated via UV-vis DRS. As illustrated in Figure 6a, CeO_2_ exhibits intrinsic absorption in the ultraviolet region (<400 nm), which is predominantly ascribed to the charge transfer from the O 2p valence band to the Ce 4f conduction band with an absorption edge at approximately 424 nm [40]. In contrast, after the incorporation of In_2_O_3_, the absorption edge of the xIn_2_O_3_-CeO_2_ composite shifts to around 448 nm. Furthermore, the visible-light absorption in the range of 400–800 nm is significantly enhanced. These results indicate that In_2_O_3_-doping effectively improves the inherent weak visible-light response of CeO_2_ [41]. The expansion of the light absorption range and the enhancement of light-harvesting capacity enable the catalyst to utilize more solar energy during the photothermal catalytic CO_2_ hydrogenation process. As shown in Figure 6b, CeO_2_ exhibits a relatively wide band gap (Eg) of 3.07 eV. Upon In_2_O_3_ incorporation, the Eg of 3% In_2_O_3_-CeO_2_ catalyst narrows to 2.98 eV. This narrowed band gap effectively extends the light absorption range, boosting visible-light harvesting and photogenerated charge carrier production.

The flat band potentials of CeO_2_ and xIn_2_O_3_-CeO_2_ were determined from Mott–Schottky plots, shown in Figure 6c. It is observed that the slopes of CeO_2_ and xIn_2_O_3_ are positive, exhibiting their n-type semiconductor characteristics [42]. Considering that the conduction band potential (E_CB_) of an n-type semiconductor is empirically positioned 0.1 eV more negative than its flat-band potential [43], the calculated E_CB_ values are −0.35 eV (CeO_2_), −0.75 eV (1% In_2_O_3_-CeO_2_), −1.53 eV (3%In_2_O_3_-CeO_2_), −1.33 eV (5% In_2_O_3_-CeO_2_) and −0.97 eV (8% In_2_O_3_-CeO_2_). Given that the standard reduction potential for CO_2_/CO is −0.53 eV vs. NHE, a more negative E_CB_ potential is thermodynamically favorable for CO_2_ reduction [44]. On the basis of the equation (E_g_ = E_VB_ − E_CB_), the valence band (E_VB_) edge positions of CeO_2_ and xIn_2_O_3_ were obtained. The E_VB_ positions of CeO_2_ and the optimized 3%In_2_O_3_-CeO_2_ were successfully calculated as 2.72 eV and 1.45 eV, respectively. These results collectively demonstrate that 3%In_2_O_3_-CeO_2_ facilitates a more efficient utilization of photogenerated electron–hole pairs, leading to enhanced photothermal catalytic activity and a more favorable thermodynamic environment for CO_2_ reduction. Figure 6d presents the electrochemical impedance spectroscopy (EIS) Nyquist plots of CeO_2_ and the xIn_2_O_3_-CeO_2_ catalysts. It is clearly observed that 3%In_2_O_3_-CeO_2_ exhibits the smallest Nyquist arc radius, signifying that it possesses the minimum charge-transfer resistance and consequently the boosted charge-transfer efficiency [45]. Such characteristics facilitate the efficient separation of photogenerated charges and the migration of carriers within 3%In_2_O_3_-CeO_2_, thereby effectively suppressing the recombination of electron–hole pairs.

To further investigate the photo-response intensity of the materials, transient photocurrent response tests were conducted (Figure 6e). Under illumination conditions, 3%In_2_O_3_-CeO_2_ displays a stable and reproducible photocurrent response with the highest photocurrent density among all the prepared catalysts, revealing its optimum photo induced charge separation efficiency. The electrochemical properties of the catalysts were further investigated by cyclic voltammetry (CV). Figure 6f displays the CV curves of xIn_2_O_3_-CeO_2_ and CeO_2_. As illustrated, the differences in the onset reduction potentials among the samples are not significant. However, 3%In_2_O_3_-CeO_2_ exhibits a higher current response throughout the entire scanning potential range, indicating that the introduction of In effectively enhances its electrochemical activity. Furthermore, a more obvious oxidation peak is observed during the reverse potential scan for 3%In_2_O_3_-CeO_2_. This observation is consistent with the excellent photothermal catalytic performance mentioned previously.

PL and TRPL were useful for investigating the charge separation and recombination behavior on CeO_2_ and 3%In_2_O_3_-CeO_2_. The TRPL corresponding fitting parameters are provided in Table S7. As shown in Figure 6f, CeO_2_ and 3%In_2_O_3_-CeO_2_ show broad defect-related emissions at 420–470, 490–530, and 600–620 nm, arising from surface O_v_, Ce^3+^ species, and Ce 4f-O 2p defect-state transitions [33]. The significantly quenched PL intensity of 3% In_2_O_3_-CeO_2_ suggests that In_2_O_3_ effectively suppresses radiative recombination of photogenerated charge carriers. As shown in Figure 6h,i, the average lifetime decreases from 2.70 ns for CeO_2_ to 2.31 ns for 3%In_2_O_3_-CeO_2_, which suggests that the radiative recombination pathway is effectively competed by a faster charge transfer process [46].

**Figure 7:**
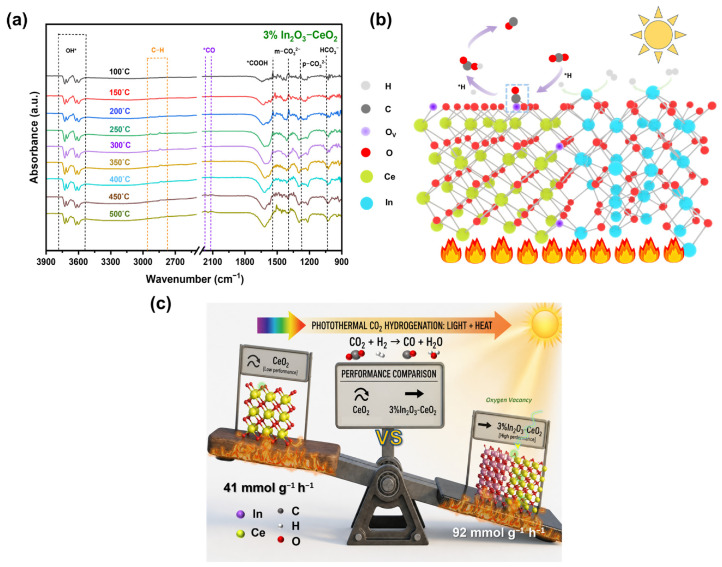
(**a**) In situ DRIFTS spectra over 3% In_2_O_3_-CeO_2_. (**b**) Photothermal catalytic RWGS mechanism over 3% In_2_O_3_-CeO_2_. (**c**) Schematic diagram of CeO_2_ and 3% In_2_O_3_-CeO_2_ on photothermal CO_2_ hydrogenation.

The key intermediates during the RWGS reaction over the 3%In_2_O_3_-CeO_2_ catalyst were investigated via in situ DRIFTS within the temperature range of 100–500 °C. As shown in Figure 7a and Figure S6, the absorption bands of the 3%In_2_O_3_-CeO_2_ and CeO_2_ observed in the range of 900–1800 cm^−1^ are predominantly assigned to carbonate intermediates. Specifically, the peaks of 3%In_2_O_3_-CeO_2_ at 1052, 1260, and 1383 cm^−1^ correspond to bicarbonate (HCO_3_^−^), polydentate (p-CO_3_^2−^), and monodentate carbonates (m-CO_3_^2−^), respectively. Furthermore, the peak at 1565 cm^−1^ is assigned to the v(COO) stretching vibration of the *COOH intermediate [47]. The simultaneous emergence of *CO peaks at 2176 and 2116 cm^−1^ confirms the formation of *CO intermediates during the RWGS reaction process, implying a reaction pathway involving the sequential conversion of CO_2_ → *COOH → *CO. Additionally, the bands at 3600–3800 cm^−1^ are attributed to surface hydroxyl groups. With increasing temperature, the intensities of these bands gradually increase, suggesting enhanced activation of CO_2_ and dissociation of H_2_ on the catalyst surface.

Based on the above analysis, a possible photothermal catalytic RWGS mechanism over 3%In_2_O_3_-CeO_2_ is proposed in Figure 7b. Initially, CO_2_ are adsorbed and activated at O_v_ sites on the CeO_2_ surface to form carbonate species. Meanwhile, H_2_ are dissociated into active hydrogen species on the In-related active sites. Subsequently, the adsorbed carbonate intermediates react with active hydrogen species to generate *COOH intermediates, which are further converted into *CO species through C-O bond cleavage, finally leading to the release of CO products. A schematic diagram of CeO_2_ and 3%In_2_O_3_-CeO_2_ on photothermal CO_2_ hydrogenation is shown in Figure 7c.

## 4. Conclusions

In this study, xIn_2_O_3_-CeO_2_ composite catalysts were successfully synthesized via the impregnation method. Experimental results demonstrate that 3% In_2_O_3_-CeO_2_ exhibits the optimal photothermal catalytic hydrogenation performance, achieving a CO_2_ reduction yield of 92.35 mmol g^−1^ h^−1^ with 100% CO selectivity. Characterization analysis reveals that In_2_O_3_ doping not only introduces abundant O_v_ to facilitate CO_2_ activation but also significantly enhances light absorption capacity and optimizes the energy band structure. Furthermore, the system effectively suppresses the recombination of photogenerated electron–hole pairs and extends carrier lifetimes, thereby improving charge separation and migration efficiency. In situ DRIFTS confirms that the reaction follows a carboxyl pathway (CO_2_ → *COOH → *CO → CO).

## Data Availability

The data that support the findings of this study are available from the corresponding author upon reasonable request.

## Supplementary Materials

The following supporting information can be downloaded at: https://www.mdpi.com/article/10.3390/nano16140872/s1, Figure S1: Photo of Beijing Perfect-light PLR-RVTF-POB fixed-bed reactor for CO_2_ hydrogenation; Figure S2: (a) XRD pattern, (b) N_2_ adsorption–desorption isotherm, (c) pore size distribution and (d) Raman spectrum of Ce-BTC; Figure S3: (a) TEM and HRTEM of 3%In_2_O_3_-CeO_2_. (b) Total count distribution spectrum of 3% In_2_O_3_-CeO_2_. Figure S4: CO_2_ conversion in photothermal CO_2_ hydrogenation over 3% In_2_O_3_-CeO_2_. Figure S5: Temperature evolution curves of CeO_2_ and 3% In_2_O_3_-CeO_2_ under light irradiation. Figure S6: In situ DRIFTS spectra over CeO_2_; Table S1: Amounts of In (NO_3_)_3_·5H_2_O and corresponding mother liquor volumes for different samples; Table S2: Different element distribution of 3%In_2_O_3_-CeO_2_; Table S3: wt% of In in 3%In_2_O_3_-CeO_2_ from ICP-OES. Table S4: Surface area, pore volume, average pore size of CeO_2_ and 3%In_2_O_3_-CeO_2_ from BET. Table S5: Atomic percent of element in 3%In_2_O_3_-CeO_2_ from XPS. Table S6: Comparison study on catalytic performance of different catalysts towards photothermal CO_2_ hydrogenation. Table S7: TRPL fitting data of CeO_2_ and 3%In_2_O_3_-CeO_2_.

## References

[1] Kar S., Kim D., Bin Mohamad Annuar A., Sarma B.B., Stanton M., Lam E., Bhattacharjee S., Karak S., Greer H.F., Reisner E. (2025). Direct air capture of CO_2_ for solar fuel production in flow. Nat. Energy.

[2] Zhang Z., Dong H., Zhou D., Tang K., Ma Y., Han T., Lei J., Huang S., Zhang X., Tang L. (2026). Electron beam- induced defect engineering construction in MIL-68(In) for enhanced CO_2_ photoreduction: Unravelling organic framework defects. J. Colloid Interface Sci..

[3] (2025). CO_2_ hydrogenation. Nat. Synth..

[4] Ye R., Ding J., Reina T.R., Duyar M.S., Li H., Luo W., Zhang R., Fan M., Feng G., Sun J. (2025). Design of catalysts for selective CO_2_ hydrogenation. Nat. Synth..

[5] Grim R.G., Badgett A., Braunecker W.A., Guarnieri M.T., Habas S.E., Hahn C., Neyerlin K., Prajapati A., Ruddy D.A., Walker R.Z. (2026). The Chemistry of CO_2_ Conversion: A Review. Chem. Rev..

[6] Li Y., Pei X., Wang Z.-J., Shi L., Song H., Ye J. (2024). Photothermal Catalytic CO_2_ Conversion to Value-Added Chemicals: Progress and Prospects. ACS Sustain. Chem. Eng..

[7] Li B., Ding Y., Li Q., Guan Z., Zhang M., Yang J. (2022). The photothermal effect enhance visible light-driven hydrogen evolution using urchin-like hollow RuO_2_/TiO_2_/Pt/C nanomaterial. J. Alloys Compd..

[8] Dong H., Zhang Z., Zhou D., Huang S., Lin Z., Zhang X., Zhang Z., Lei J., Liu N. (2026). Progress of photothermal/thermal catalytic CO_2_ hydrogenation by metal-modified CeO_2_. Green Chem..

[9] Montini T., Melchionna M., Monai M., Fornasiero P. (2016). Fundamentals and Catalytic Applications of CeO2-Based Materials. Chem. Rev..

[10] Huang W., An B., Chen Z., Han Y., Chen Y., Li J., Han X., Xu S., Crawshaw D., Tillotson E. (2025). Synthesis of Primary Amines via Reductive Amination of Aldehydes and Ketones Over a Ni-Doped MFM-300(Cr) Catalyst. Adv. Sci..

[11] Pan D., Wang Y., Li H., Zhang Y., Liang Q., Zhou M., Li Z., Xu S. (2024). Solar light promoted CO_2_ hydrogenation to CH_4_ over photo-thermal responsive dispersed Co on defective CeO_2_ composite derived from MOFs. Sep. Purif. Technol..

[12] Mourdikoudis S., Dutta S., Kamal S., Gómez-Graña S., Pastoriza-Santos I., Wuttke S., Polavarapu L. (2025). State-of-the-Art, Insights, and Perspectives for MOFs-Nanocomposites and MOF-Derived (Nano)Materials. Adv. Mater..

[13] Deng B., Song H., Peng K., Li Q., Ye J. (2021). Metal-organic framework-derived Ga-Cu/CeO_2_ catalyst for highly efficient photothermal catalytic CO_2_ reduction. Appl. Catal. B Environ..

[14] Kim J., Park H., Kim D., Yang S., Song S., Choi Y., Kim H., Bae J.-S., Le C.T., Kim Y.S. (2021). Bi doping stimulation on the visible-light absorption of In_2_O_3_ ceramics. J. Alloys Compd..

[15] Koley P., Shit S.C., Yoshida T., Jampaiah D., Ariga-Miwa H., Uruga T., Kaishyop J., Hosseinnejad T., Periasamy S., Gudi R.D. (2025). Metal organic framework derived In_2_O_3_/ZrO_2_ heterojunctions with interfacial oxygen vacancies for highly selective CO_2_-to-methanol hydrogenation. Nat. Commun..

[16] Yang J., Tian Z., Ren Y., Bai S., He G., Yu F., Wang F., Liu Y., Guo L. (2026). Synergistic Electron-Proton Transfer Over In_2_O_3_/CuGa0.5S Z-Scheme Heterojunction for Highly Selective CO_2_-to-CH_4_ Photoconversion. Angew. Chem. Int. Ed..

[17] Martin O., Martín A.J., Mondelli C., Mitchell S., Segawa T.F., Hauert R., Drouilly C., Curulla-Ferré D., Pérez-Ramírez J. (2016). Indium Oxide as a Superior Catalyst for Methanol Synthesis by CO_2_ Hydrogenation. Angew. Chem. Int. Ed..

[18] Wang W., Zhang Y., Wang Z., Yan J.-M., Ge Q., Liu C.-J. (2016). Reverse water gas shift over In_2_O_3_–CeO_2_ catalysts. Catal. Today.

[19] Chen B., Zeng X., Liu Y., Xiao F., Huang M., Tan K.B., Cai D., Huang J., Zhan G. (2022). Thermal decomposition kinetics of M−BTC (M = Cu, Co, Zn, and Ce) and M−BTC/Pt composites under oxidative and reductive environments. Chem. Eng. J..

[20] Jiang Y., Gao J., Zhang Q., Liu Z., Fu M., Wu J., Hu Y., Ye D. (2019). Enhanced oxygen vacancies to improve ethyl acetate oxidation over MnOx-CeO_2_ catalyst derived from MOF template. Chem. Eng. J..

[21] Khade C.P., Zoting K.R., Mandawade A.S., Dighe A.K., Jadhav O.G., Dinde P.P., Marimuthu R., Ghule B.G., Gholap H.M. (2026). Charge transfer mechanism of CO adsorption on the surface of CeO_2_ and Mn-Doped CeO_2_ nanoparticles: An experimental and ab initio study. Appl. Surf. Sci..

[22] Pan Y.Q., Zhang Z.Y., Li W.T., Xu B.Y., Yao J.L., Liu D.K., Xie T. (2025). Pt–In_2_O_3_/CeO_2_ for photothermal catalytic CO_2_ hydrogenation to methanol under atmospheric pressure: Targeted regulation of In_2_O_3_ on key intermediate species. Int. J. Hydrogen Energy.

[23] Zhu L., Liu Y., Gao Y., Ding N., Wang D., Long L., Wang B., Lang J., Vovk E.I., Yang Y. (2025). Mechanistic Understanding of Dissociated Hydrogen in Cu/CeO_2_-Catalyzed Methanol Synthesis. ACS Appl. Mater. Interfaces.

[24] Zhang L., Zhou G., Chen G., Wang H., Zhao Q., Yin W., Yi J., Zhu X., Wang X., Ning X. (2024). Bimetallic NiCu catalyst derived from spent MOF adsorbent for efficient photocatalytic CO_2_ reduction. Chem. Eng. J..

[25] Shibeshi P.T., Parajuli D., Murali N. (2022). Study of Fe-doped and glucose-capped CeO2 nanoparticles synthesized by co-precipitation method. Chem. Phys..

[26] Penkova A., Laguna O.H., Centeno M.A., Odriozola J.A. (2012). CO-Induced Morphology Changes in Zn-Modified Ceria: A FTIR Spectroscopic Study. J. Phys. Chem. C.

[27] Fan L., Wang K., Xu K., Liang Z., Wang H., Zhou S.-F., Zhan G. (2020). Structural Isomerism of Two Ce-BTC for Fabricating Pt/CeO_2_ Nanorods toward Low-Temperature CO Oxidation. Small.

[28] Xu L., Xu J., Shen B., Yu T. (2025). Photothermal enhanced exciton dissociation and protonation reaction over Ru/CeO_2_ single-atom catalyst for efficient CO_2_ methanation. Chem. Eng. J..

[29] Bai X., Wu Y., Sun Y., Guo X., Niu Y., Meng T., Zhang H., He J. (2026). Engineering oxygen vacancies in Ni/CeO_2_-nanorods via MOF templating for high-efficiency photothermal CO_2_ hydrogenation. Chem. Eng. J..

[30] Yang C., Pei C., Luo R., Liu S., Wang Y., Wang Z., Zhao Z.-J., Gong J. (2020). Strong Electronic Oxide–Support Interaction over In_2_O_3_/ZrO_2_ for Highly Selective CO_2_ Hydrogenation to Methanol. J. Am. Chem. Soc..

[31] Sharma P., Ho P.H., Shao J., Creaser D., Olsson L. (2023). Role of ZrO_2_ and CeO_2_ support on the In_2_O_3_ catalyst activity for CO_2_ hydrogenation. Fuel.

[32] Xu Q.-J., Jiang J.-W., Wang X.-F., Duan L.-Y., Guo H. (2023). Understanding oxygen vacant hollow structure CeO_2_@In_2_O_3_ heterojunction to promote CO_2_ reduction. Rare Met..

[33] Wang Q., Chen Y., Liu X., Li L., Du L., Tian G. (2021). Sulfur doped In_2_O_3_-CeO_2_ hollow hexagonal prisms with carbon coating for efficient photocatalytic CO_2_ reduction. Chem. Eng. J..

[34] Lu B., Quan F., Sun Z., Jia F., Zhang L. (2019). Photothermal reverse-water-gas-shift over Au/CeO_2_ with high yield and selectivity in CO_2_ conversion. Catal. Commun..

[35] Yang Z., Zeng M., Wang K., Yue X., Chen X., Dai W., Fu X. (2022). Visible light-assisted thermal catalytic reverse water gas reaction over Cu-CeO_2_: The synergistic of hot electrons and oxygen vacancies induced by LSPR effect. Fuel.

[36] Zhu Z., Zhou J., Li Q., Liu Z., Deng Q., Zhou Z., Li C., Fu L., Zhou J., Li H. (2024). Preparation of heterostructured Cu-CeO_2_/SrTiO_3_ catalysts by rapid plasma exsolution for photothermal reverse water gas shift reaction. J. CO_2_ Util..

[37] Zhao J., Yang Q., Shi R., Waterhouse G.I.N., Zhang X., Wu L.Z., Tung C.H., Zhang T. (2020). FeO-CeO_2_ nanocomposites: An efficient and highly selective catalyst system for photothermal CO_2_ reduction to CO. NPG Asia Mater..

[38] Jia Z., Ning S., Tong Y., Chen X., Hu H., Liu L., Ye J., Wang D. (2021). Selective Photothermal Reduction of CO_2_ to CO over Ni-Nanoparticle/N-Doped CeO_2_ Nanocomposite Catalysts. ACS Appl. Nano Mater..

[39] Ren Y., Si Y., Du M., You C., Zhang C., Zhu Y.H., Sun Z., Huang K., Liu M., Duan L. (2024). Photothermal Synergistic Effect Induces Bimetallic Cooperation to Modulate Product Selectivity of CO_2_ Reduction on Different CeO_2_ Crystal Facets. Angew. Chem. Int. Ed..

[40] Guo X., Wu Y., Shao Y., Zhou S., Song H., Izumi Y., Deng L., Wang W., He J., Zhang H. (2025). Dual Active Sites of Embedded Ni and Surface Frustrated Lewis Pairs on CeO_2_(110) for Efficient Photocatalytic CO_2_ Methanation. ACS Nano.

[41] Qi Y., Jiang J., Liang X., Ouyang S., Mi W., Ning S., Zhao L., Ye J. (2021). Fabrication of Black In_2_O_3_ with Dense Oxygen Vacancy through Dual Functional Carbon Doping for Enhancing Photothermal CO_2_ Hydrogenation. Adv. Funct. Mater..

[42] Zhu C., Wei X., Li W., Pu Y., Sun J., Tang K., Wan H., Ge C., Zou W., Dong L. (2020). Crystal-Plane Effects of CeO_2_{110} and CeO_2_{100} on Photocatalytic CO_2_ Reduction: Synergistic Interactions of Oxygen Defects and Hydroxyl Groups. ACS Sustain. Chem. Eng..

[43] Zhang Z., Huang W., Dong H., Cui H., Zhou D., Lin Z., Hu B., Zhang X., Huang S., Tang L. (2026). Self-Tuned Ligand-to-Metal–Metal Charge Transfer in Node-Engineered Al-Doped NH_2_-UiO-66 Boosts CO_2_ Photoreduction Activity. Adv. Funct. Mater..

[44] Choi C., Zhao F., Hart J.L., Gao Y., Menges F., Rooney C.L., Harmon N.J., Shang B., Xu Z., Suo S. (2023). Synergizing Electron and Heat Flows in Photocatalyst for Direct Conversion of Captured CO_2_. Angew. Chem. Int. Ed..

[45] Huang Q., Xia G.-J., Huang B., Xie D., Wang J., Wen D., Lin D., Xu C., Gao L., Wu Z. (2024). Activating lattice oxygen by a defect-engineered Fe_2_O_3_–CeO_2_ nano-heterojunction for efficient electrochemical water oxidation. Energy Environ. Sci..

[46] Xu F., Zhao F., Deng X., Zhang J., Zhang J., Ai C., Yu J., García H. (2025). Integrating S-scheme photocatalysis with tandem carbonylation: A green and scalable strategy for CO_2_ valorization. Nat. Commun..

[47] Zhou D., Zhang Z., Dong H., Liu Y., Hu B., Huang S., Zhang X., Lei J., Liu N. (2025). In situ electronic modulation of g-C_3_N_4_/UiO66 composites via N species functionalized ligands for enhanced photocatalytic CO_2_ reduction. Sep. Purif. Technol..

