# Supplementary material for: MOF-Derived In2O3-CeO2 Composite Catalyst with Abundant Oxygen Vacancies for Photothermal CO2 Reduction
Source: Nanomaterials (Basel). 2026 Jul 15;16(14):872. doi: 10.3390/nano16140872 (PMC13415073; doi:10.3390/nano16140872)
Supplement: Supplementary file 1 [file nanomaterials-16-00872-s001.zip › nanomaterials-4400112-supplementary.pdf]

MOF-Derived In_2O_3-CeO_2 Composite Catalyst with Abundant Oxygen Vacancies for Photothermal CO_2 Reduction

Huiqing Dong [1], Siyu Huang [1], Haopeng Cui [1], Ziyi Zhang [1,2], Dongxu Zhou [1], Weikai Huang [1], Xiaodong Zhang [1,3], Zhongxiao Zhang [3,4], Jianqiu Lei [5] and Ning Liu [1,3,*]

1 School of Environment and Architecture, University of Shanghai for Science and Technology, Shanghai 200093, China; dhq200223@163.com (H.D.); 13958318481@163.com (S.H.); m15930993445@163.com (H.C.); 18067069811@163.com (Z.Z.); zdx17730040824@163.com (D.Z.); 15021163085@163.com (W.H.); fatzhxd@126.com (X.Z.)
2 Beijing National Laboratory for Molecular Sciences, CAS Key Laboratory of Colloid, Interface and Chemical Thermodynamics, Institute of Chemistry, Chinese Academy of Science, Beijing 100190, China
3 Shanghai Noncarbon Energy Conversion and Utilization Institute, Shanghai 200240, China; zhzhx222@163.com
4 College of Smart Energy, Shanghai Jiao Tong University, Shanghai 200240, China
5 Shanghai Institute of Optics and Fine Mechanics, Chinese Academy of Sciences, Shanghai 201800, China; ljqlei@163.com
* Correspondence: liuning6910@163.com or liuning@usst.edu.cn

The detailed calculation formula for ICP-OES is provided below:

$$C_x = \frac{C_0 * f * V_0 * 10^{-3}}{m * 10^{-3}} = \frac{C_1 * V_0 * 10^{-3}}{m * 10^{-3}} \tag{S1}$$

$$W(\%) = \frac{C_x}{10^6} * 100\% \tag{S2}$$

where m_0 represents the mass of the digested sample in g, V_0 is the final constant volume of the sample digestion solution in mL, f is the dilution factor, C_0 is the elemental concentration of the tested solution in mg L^{-1}, C_1 is the elemental concentration of the original digestion solution in mg L^{-1}, C_x is the final elemental content in the sample expressed in mg kg^{-1}, and W is the final elemental content expressed as a percentage.

The detailed calculation formula for XPS is provided below:

the corrected intensity of each element was obtained by normalizing the integrated peak area using the corresponding relative sensitivity factor according to the following equation:

$$Ii^{corrected} = \frac{Ai}{SFi} \tag{S3}$$

where (Ai) is the integrated peak area of element (i), and (SFi) is the corresponding relative sensitivity factor. The atomic percentage of each element was then calculated from the normalized peak area, Area(N), using the following equation:

$$Atomic\ \% = \frac{Ii^{corrected}}{\sum Ii^{corrected}} \tag{S4}$$

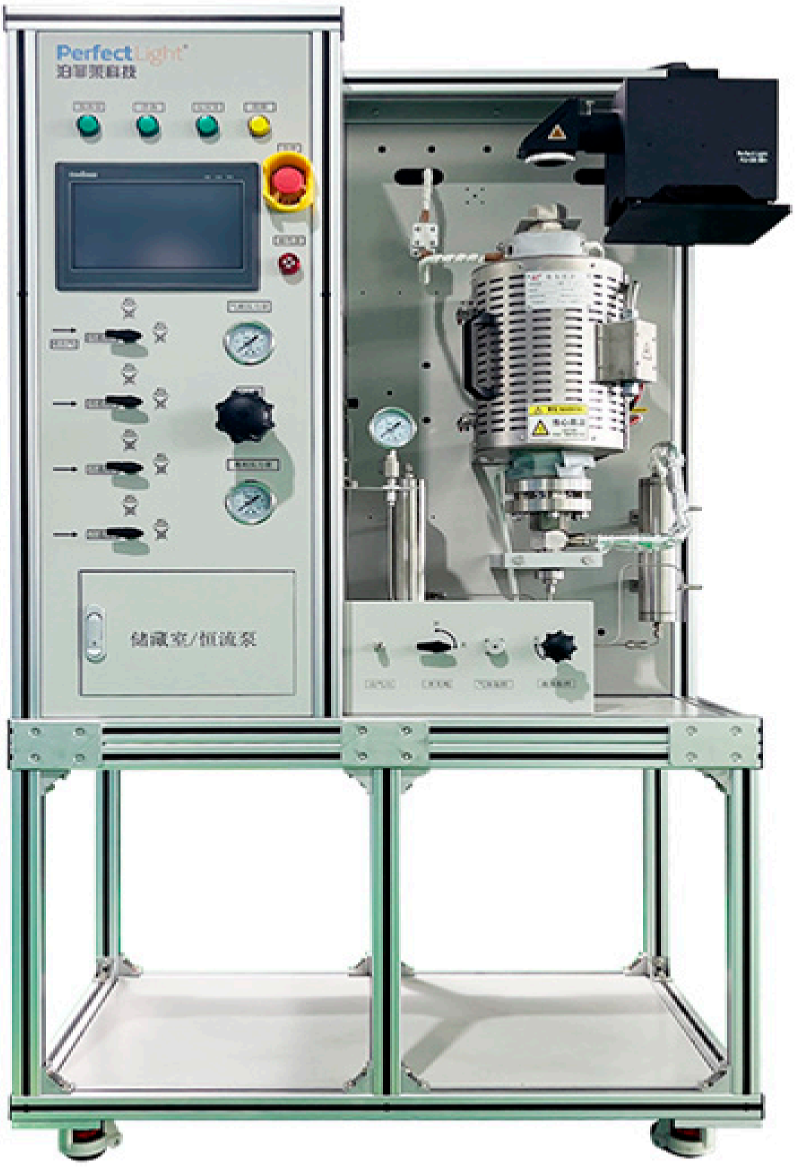

Figure S1. Photo of Beijing Perfect-light PLR-RVTF-POB fixed-bed reactor for CO_2 hydrogenation.

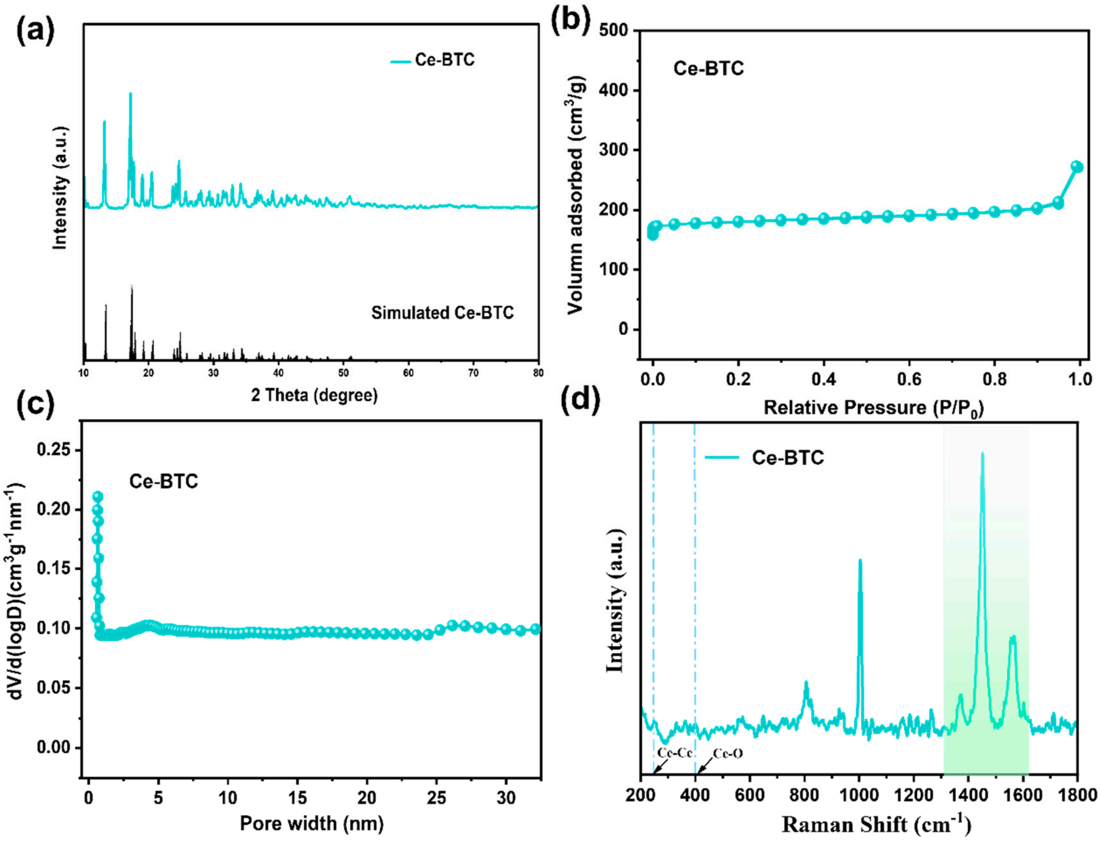

Figure S2. (a) XRD pattern, (b) N_2 adsorption-desorption isotherm, (c) Pore size distribution and (d) Raman spectrum of Ce-BTC.

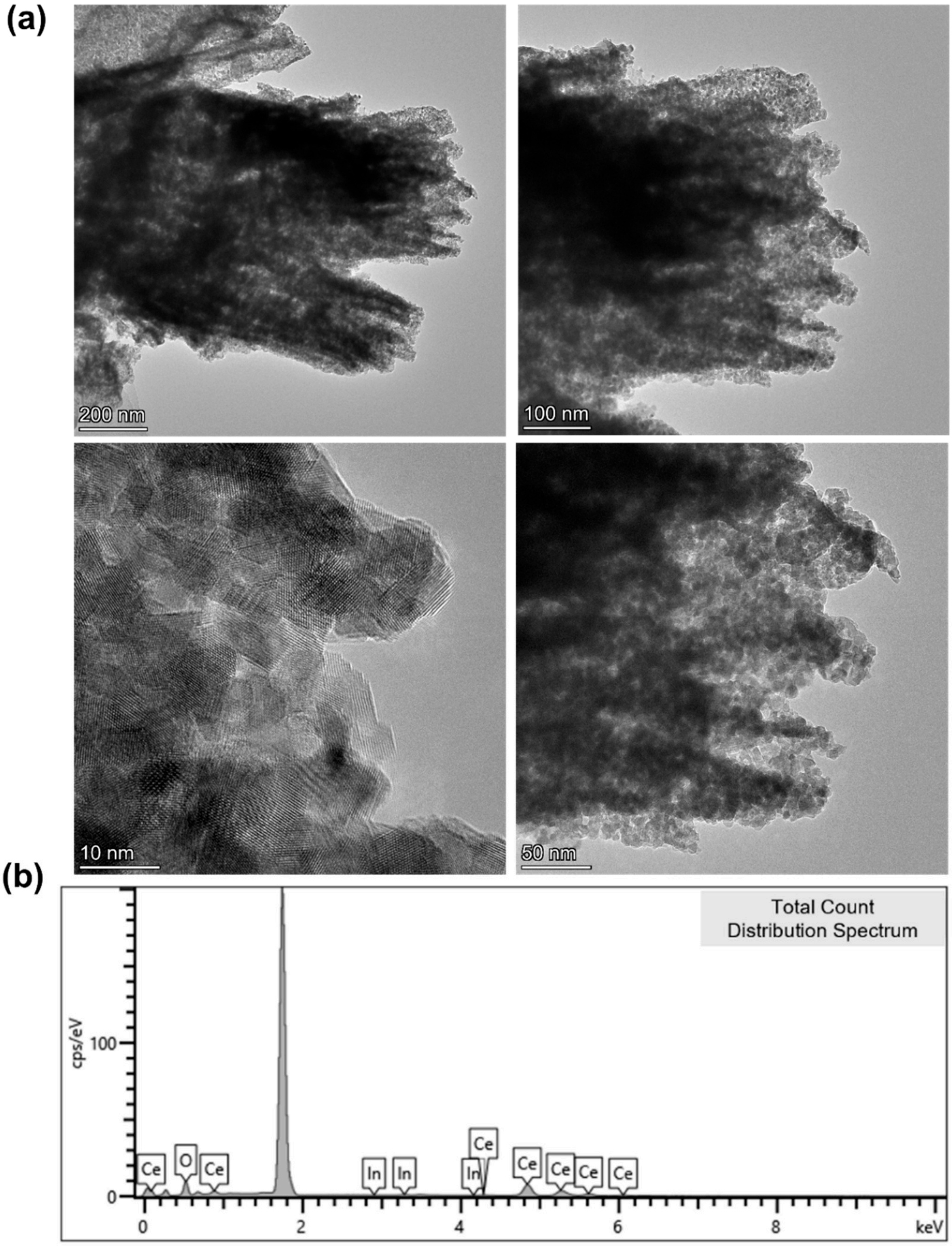

Figure S3. (a) TEM and HRTEM of 3%In_2O_3-CeO_2. (b) Total count distribution spectrum of 3% In_2O_3-CeO_2.

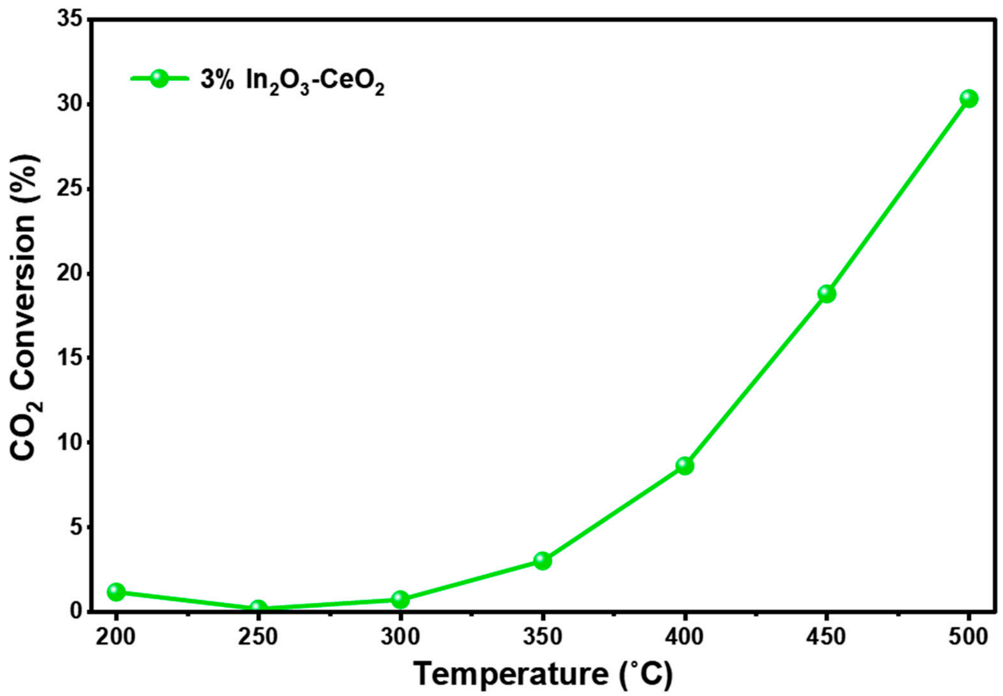

Figure S4. CO_2 conversion in photothermal CO_2 hydrogenation over 3% In_2O_3-CeO_2.

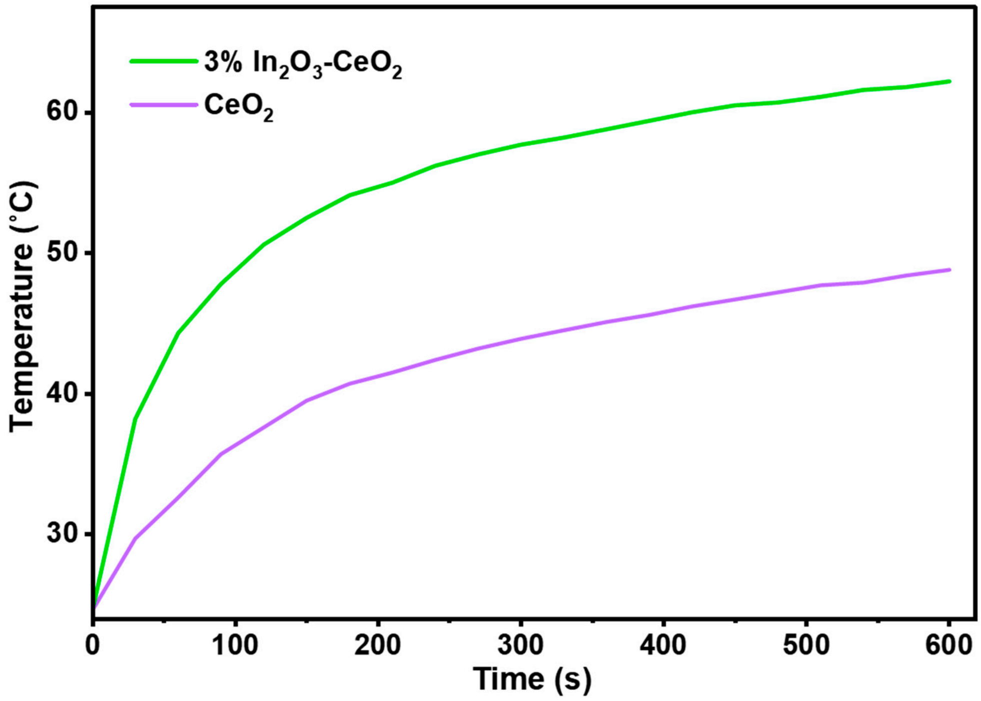

Figure S5. Temperature evolution curves of CeO_2 and 3% In_2O_3-CeO_2 under light irradiation.

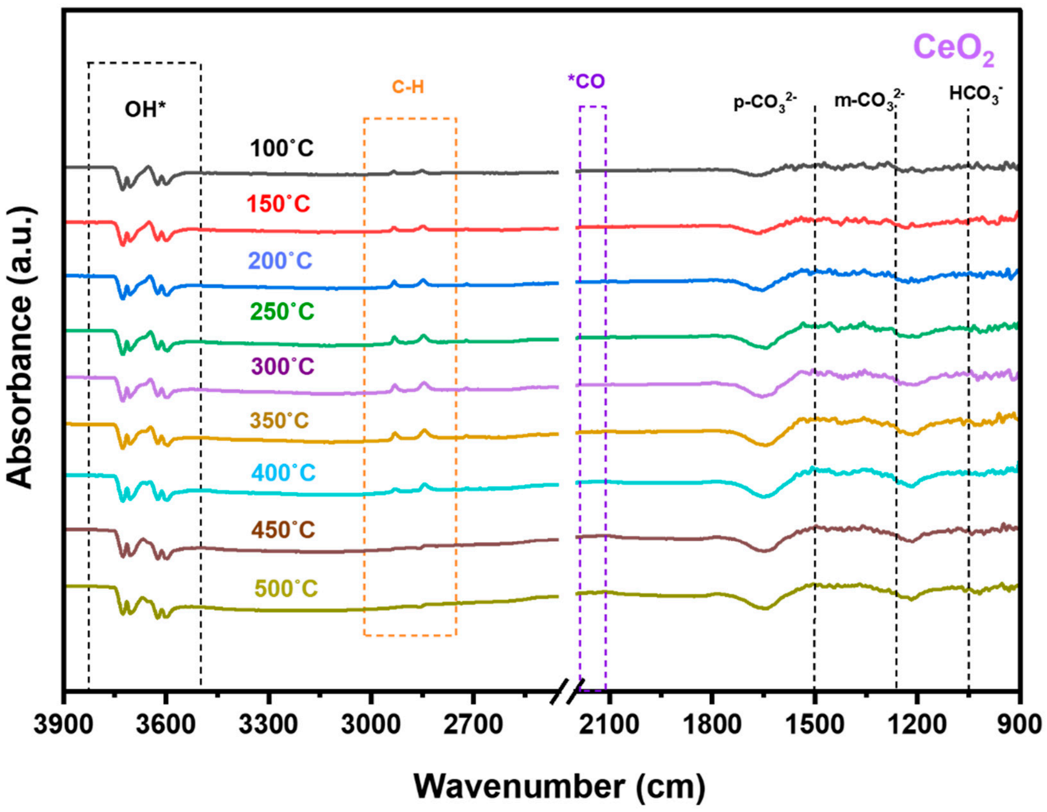

Figure S6. In-situ DRIFTS spectra over CeO_2.

Table S1. Amounts of In $(NO_3)_3 \cdot 5H_2O$ and corresponding mother liquor volumes for different samples.

In %	In $(NO_3)_3 \cdot 5H_2O$ /mg	Mother liquor / mL
1%	15.2	1.52
3%	46.8	4.68
5%	79.6	7.96
8%	131.4	13.14

Table S2. Different element distribution of 3%In_2O_3-CeO_2.

Element	Wt%	Wt% Sigma
O	29.58	0.16
In	1.88	0.09
Ce	68.54	0.19

Table S3. wt% of In in 3%In_2O_3-CeO_2 from ICP-OES

Element	V_0 (mL)	m_0 (g)	C_0 (mg/L)	f	C_1(mg/L)	C_x (mg/kg)	W (%)
Ce	25	0.0223	6.7252	100	672.5204	753946.68	75.3946
In	25	0.0223	2.1090	10	21.0904	23643.976	2.3643

Table S4. Surface area, pore volume, average pore size of CeO_2 and 3%In_2O_3-CeO_2 from BET.

Samples	S_{BET} (m^2/g)	Pore volume (cc/g)	Average pore size (nm)
CeO_2	111.43	0.262	3.415
3%In_2O_3-CeO_2	83.79	0.285	3.417

Table S5. Atomic percent of element in 3%In_2O_3-CeO_2 from XPS

Name	Area (P) CPS. eV	SF ALTHERMO1	Area (N) TPP-2M	Atomic %	Atomic % excluding C
C1s	80819.19	1	1133.28	30.7	-
In3d	73940.86	32.235	35.86	0.97	1.4
O1s	334015.69	2.881	1933.1	52.37	75.57
Ce3d	1545939.25	61.447	588.93	15.96	23.03
Total	-	-	3691.17	100	100

Table S6. Comparison study on catalytic performance of different catalysts towards photothermal CO_2 hydrogenation

Catalysts	CO yield (mmol g^{-1} h^{-1})	Selectivity (%)	References
Au-CeO_2	0.64	100	[34]
Cu-CeO_2	-	100	[35]
Cu-CeO_2/$SrTiO_3$	11.32	99.95	[36]
FeO-CeO_2	19.61	99.87	[37]
Ni/N-CeO_2	20.9	100	[38]
Pt/CeO_2	29.24×10^{-3}	100	[39]
In_2O_3-CeO_2	92.35	100	This work

Table S7. TRPL fitting data of CeO_2 and 3%In_2O_3-CeO_2

Model	ExpDec1	
Equation	y = A1*exp(-x/τ1)	
Sample	CeO_2	3% In_2O_3-CeO_2
A1	26.19	27.57
τ1(ns)	2.70	2.31
χ2	1.115	1.104
